# Depression after low-energy fracture in older women predicts future falls: a prospective observational study

**DOI:** 10.1186/1471-2318-11-73

**Published:** 2011-11-07

**Authors:** Martha van den Berg, Noortje A Verdijk, Geraline L Leusink, Colette JM Wijnands-van Gent, Arnold C Romeijnders, Victor JM Pop, Joop PW van den Bergh

**Affiliations:** 1 Tilburg University, CoRPS - Center of Research on Psychology in Somatic diseases, PO Box 90153, 5000 LE Tilburg, the Netherlands; 2 Diagnostiek voor U, PO Box 2406, 5600 CK Eindhoven, the Netherlands; 3 Stichting Severinus, PO Box 6666, 5500 MA Veldhoven, the Netherlands; 4 PoZoB, Coordination Centre of Practice Nurses for South East Netherlands, PO Box 312, 5500 AH Veldhoven, the Netherlands; 5 VieCuri Medical Centre Noord-Limburg, Department of Internal Medicine, PO Box 1926, 5900 BX Venlo, the Netherlands; 6 Maastricht University/Nutrim, Faculty of Health Medicine and Life Science, Department of Internal Medicine, PO Box 616, 6200 MD Maastricht, the Netherlands

## Abstract

**Background:**

Falls are one of the main causes of fractures in elderly people and after a recent fracture, the risk of another fall is increased, resulting in subsequent fracture. Therefore, risk factors for future falls should be determined. We prospectively investigated the relationship between depression and the incidence of falls in post-menopausal women after a low-energy fracture.

**Methods:**

At baseline, 181 women aged 60 years and older who presented with a recent low-energy fracture were evaluated at the fracture and osteoporosis outpatient clinics of two hospitals. As well as clinical evaluation and bone mineral density tests, the presence of depression (measured using the Edinburgh Depression Scale, EDS, depression cut-off > 11) and risk factors for falling were assessed. During two years of follow-up, the incidence of falls was registered annually by means of detailed questionnaires and interviews.

**Results:**

Seventy-nine (44%) of the women sustained at least one fall during follow-up. Of these, 28% (*n *= 22) suffered from depression at baseline compared to 10% (*n *= 10) of the 102 women who did not sustain a fall during follow-up (*Χ*^2 ^= 8.76, df = 1, *p *= .003). Multiple logistic regression showed that the presence of depression and co-morbidity at baseline were independently related to falls (OR = 4.13, 95% CI = 1.58-10.80; OR = 2.25, 95% CI = 1.11-4.56, respectively) during follow-up.

**Conclusions:**

The presence of depression in women aged 60 years and older with recent low-energy fractures is an important risk factor for future falls. We propose that clinicians treating patients with recent low-energy fractures should anticipate not only on skeletal-related risk factors for fractures, but also on fall-related risk factors including depression.

## Background

Falls are a major problem in older adults. The incidence increases with age and is higher in women than in men [1,2]. Furthermore, falls are among the main causes of diminished functioning and hospitalisation [3,4]. In 2002, it was estimated that, worldwide, 391,000 people of all ages died of injury-related falls in that year [5]. The costs of non-fatal fall injuries among adults over 64 years of age in the US were estimated at $19 billion in 2000 [6].

Up to 70% of low-energy fractures (defined as resulting from a fall from standing height or lower) are caused by falls [7,8]. Furthermore, it has been shown that 19% of women with a recent low-energy fracture reported another fall within three months of that fracture [9]. Falls are a strong and independent risk factor for fractures in elderly people [10]. Therefore, after age and bone mineral density (BMD), the number of falls during the past 12 months was included in the recently developed Garvan nomogram that can be used for the calculation of absolute five- and 10-year fracture risk [11,12]. For the prevention of fractures, attention should not only be focussed on the prevention and treatment of low BMD, but also on the prevention of falls.

Several risk factors for falls in older people have been studied. In a recent systematic review, a total of 31 risk factors were distinguished, assessed by at least five studies [1]. Of these, age, female sex, a history of falls, co-morbidity and the use of medication were among the factors most frequently studied, and which had the greatest impact on future falls. Moreover, depression was found to have a negative effect on falls [1].

Depression (high depressive symptom scores as well as syndromic depression) has been described as a potential risk factor for falls in various samples and settings [13-16]. The prevalence of high depressive symptom scores in elderly Dutch women has been estimated to be 17% [17]. In Dutch primary care, 19% of women aged over 55 years suffer from high depressive symptomatology [18]. Furthermore, it has been shown that depressed patients suffer from poorer recovery after fracture [19,20].

To the best of our knowledge, the relationship between depression and falls in patients with a history of recent low-energy fracture has not yet been studied. Therefore, the current study investigated the occurrence of depression and falls during a two-year follow-up period in post-menopausal women with a recent low-energy fracture.

## Methods

### Subjects

**Figure 1:**
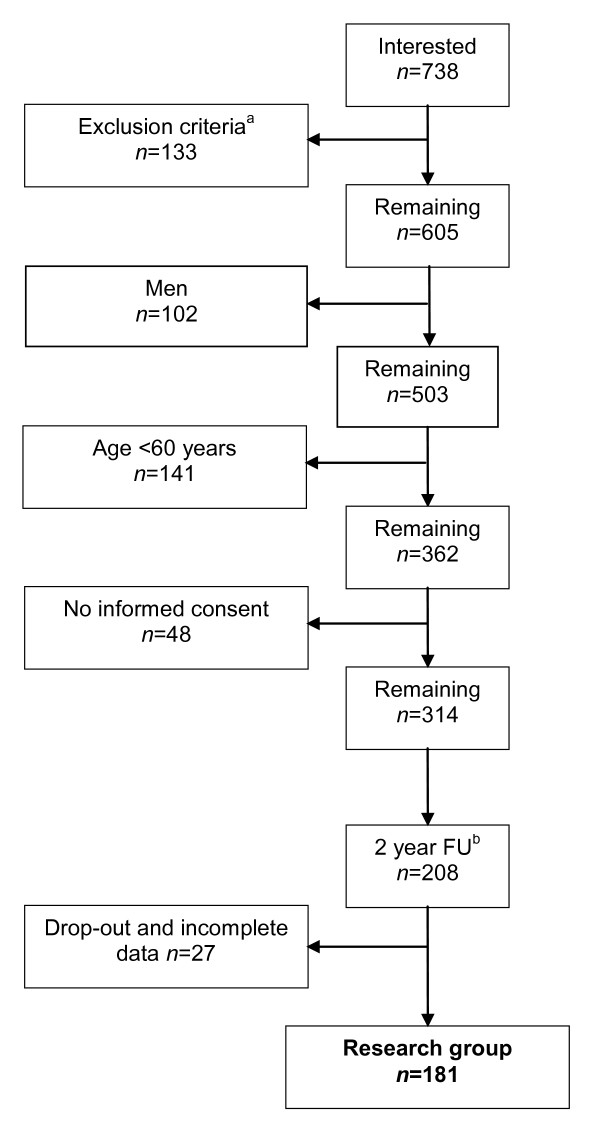
**Flowchart of participants included in the current two-year follow-up study**. ^a ^Insufficient knowledge of the Dutch language n = 6, inadequate cognitive abilities n = 12, history of fracture > 3 months earlier n = 115.^b ^FU = Follow-up

The current study was part of a larger project on the development of an osteoporosis care management programme in primary care [21]. Between October 2006 and July 2008, primary care patients who visited the fracture and osteoporosis (F&O) outpatient clinics of two hospitals in the south-east of the Netherlands after suffering from low-energy fracture, were informed about the project. During the period of inclusion, 738 patients aged 50 years and older who visited the F&O outpatient clinics, were interested in participating (Figure 1). After primary fracture care, all patients were invited to undergo BMD measurement and further clinical evaluation by a specialised nurse.

Patients with insufficient knowledge of the Dutch language (*n *= 6), inadequate cognitive abilities (*i.e*., pre-dementia, *n *= 12), or a fracture that had occurred more than three months previously (*n *= 115), were excluded for the current study. Moreover, all men as well as women younger than 60 years of age were also excluded. A total of 362 women were eligible for participation. Ultimately, 314 women (87%) provided written informed consent and 208 completed the two-year follow-up (Figure 1).

At baseline, as well as regular F&O assessment, all the patients completed a set of standardised questionnaires for the assessment of depressive symptoms and the presence of risk factors for falling. During follow-up, the incidence of falling was registered annually by means of detailed questionnaires and interviews. Since 27 of the women returned incomplete questionnaires, the final sample for data analysis includes 181 women. All women were advised to use adequate calcium and vitamin D supplementation. Women with osteoporosis were referred to their general practitioner for treatment with anti-osteoporosis medication. The study was approved by the medical ethical committee of the Máxima Medical Centre Veldhoven, the Netherlands, and was carried out in accordance with the Declaration of Helsinki.

### Measurements

#### Demographic and fracture characteristics

Demographic characteristics (age, marital status and education level) were collected using self-report forms (Table 1). The demographic characteristics of the women participating in this study were similar to those of the total female population visiting the F&O clinics (data not shown). Information regarding fracture type was provided by the F&O clinics, and was classified according to Center *et al*. [22] into hip fractures, major fractures (vertebra, pelvis, distal femur, proximal tibia, multiple rib, and proximal humerus), minor fractures (all remaining osteoporotic fractures, excluded fingers and toes), and finger and toe fractures.

**Table 1:** Baseline characteristics of 181 women with a recent fracture who did or did not sustain a fall during the two-year follow-up period

Variable		Did not fall (*n *= 102)	Fell (*n *= 79)
*Demographic characteristics*			
Age (mean, SD)*		**67.98 (5.73)**	**70.05 (6.70)**
Marital status	Married/living together	70 (69%)	48 (61%)
	Single/divorced/widowed	32 (31%)	31 (39%)
Education	Elementary school	59 (58%)	40 (51%)
	Middle school/high school	37 (36%)	33 (42%)
	College/University	6 (6%)	6 (8%)
*BMD measurements*			
BMD femoral neck (mean, SD)		0.68 (0.11)	0.66 (0.11)
BMD lumbar spine (mean, SD)		0.87 (0.15)	0.84 (0.23)
WHO classification	Osteoporosis	48 (47%)	42 (53%)
	Osteopenia	32 (31%)	26 (33%)
	Normal BMD	22 (22%)	11 (14%)
Type of fracture according to Center [22]	Hip	11 (11%)	8 (10%)
	Major	24 (24%)	15 (19%)
	Minor	55 (54%)	43 (54%)
	Fingers/toes	12 (12%)	13 (16%)

* *p *< .05

#### Bone Mineral Density

BMD was measured using a Hologic W Dual energy X-ray Absorptiometry (DXA) system. In accordance with the World Health Organization (WHO) classification, osteoporosis was defined as a T-score ≤ -2.5 SD in the spine and/or femoral neck, osteopenia as a T-score < -1.0 and > -2.5 SD, and normal BMD as a T-score ≥ - 1.0 [23].

#### Risk factors for falling

Risk factors for falling were determined at baseline in accordance with the Dutch guidelines 'Prevention of fall incidents in the elderly' [24,25]. These guidelines were developed in 2004 by the Dutch Institute for Healthcare Improvement [24,25]. The following characteristics were assessed at baseline: age, living on one's own, ≥1 falls during the 12 months prior to inclusion, use of a walking aid, use of anti-depressants, use of sedatives, use of antihypertensives, ≥2 units of daily alcohol consumption, and physical inactivity. The existence of co-morbidity (history of stroke, urinary incontinence, osteoarthritis, rheumatic disease, diabetes and Parkinson's disease) was checked by the specialised nurse, based on the patients' medical records and information from the treating physicians. An overview of the fall-related risk factors is presented in Table 2 for women who did or did not sustain a fall during the follow-up period.

**Table 2:** The prevalence of fall-related risk factors at baseline in 181 women with a recent fracture who did or did not sustain a fall during the two-year follow-up period

Variable	Did not fall *n *= 102 (%)	Fell *n *= 79 (%)
Living on one's own	31 (30)	29 (37)
≥1 fall during 12 months prior to inclusion	77 (75)	66 (84)
Use of walking aid	16 (16)	12 (15)
Comorbidity^a^*	**47 (46)**	**55 (70)**
Use of anti-depressants	3 (3)	5 (6)
Use of sedatives	8 (8)	14 (18)
Use of antihypertensives	35 (34)	25 (32)
≥2 units of daily alcohol consumption	22 (22)	19 (24)
Physical inactivity	14 (14)	16 (20)
Depression according to the EDS^b^*	**10 (10)**	**22 (28)**

* *p *< .05

^a ^history of stroke, urinary incontinence, osteoarthritis, rheumatic disease, diabetes and Parkinson's disease

^b ^score > 11

#### Depressive symptoms

The Edinburgh Depression Scale (EDS) [26] was used to assess depressive symptoms at baseline. The EDS is a ten-item self-rating scale performed over a seven-day period with a four-point scale ranging from 0 to 3 (range 0-30). It was originally designed as the Edinburgh Postnatal Depression Scale (EPDS) for detecting postnatal depression in postpartum women [26]. The Dutch version of the EPDS has been validated showing appropriate psychometric characteristics [27]. The EPDS was later validated in a group of non-childbearing mothers, and middle-aged women, as well as in subjects aged over 55 years (men and women), and renamed the Edinburgh Depression Scale (EDS) [28-30]. The internal consistency of the EDS is good and its specificity and positive predictive value are appropriate [26-30]. Higher scores indicate the presence of more depressive symptoms. In the present study, depression was defined as an EDS score > 11.

### Statistical analyses

In order to determine differences in baseline characteristics, including the presence of depression, between women who did or did not sustain a fall during the follow-up period, independent samples t-test and Chi-square tests were used. To explore the relationship between continuous variables, Pearson correlations were calculated (two-tailed). Unadjusted ORs (*p *< 0.05, 95% CI) were calculated using single logistic regression analyses, with falls as the dependent variable. Adjusted ORs were calculated using multiple logistic regression analysis, with presence or absence of falls during the two-year follow-up period as the dependent variable, entering all risk factors into the regression analysis. Statistical analyses were performed using the IBM Statistical Package for the Social Sciences (IBM SPSS) version 18.0.

## Results

### Baseline characteristics and falls during follow-up

The mean age of the participating women was 69 years (range, 60-84) and the majority was either married or living together with a partner (65%). During the two-year follow-up period, 44% (*n *= 79) of the women sustained at least one fall; in total there were 220 fall incidents in these 79 women (mean = 2.78; SD = 2.58). Women who sustained a fall during follow-up were significantly older than those who did not (t = 2.19, *p *= 0.03; Table 1). As shown in Table 1, there was no significant difference in marital status, education, BMD of the femoral neck and the lumbar spine, WHO T-score classification, or type of fracture between the two groups.

Of the 79 women who sustained a fall during follow-up, 57 (72%) fell at least once in the first year, and 43 (54%) fell at least once during the second year. The number of women who sustained a first fall during the first year (*n *= 57) was significantly larger compared to that during the second year (*n *= 22) (*Χ*^2 ^= 18.72, df = 1, *p *< .001). However, with regard to the total number of falls (*n *= 220), there was no significant difference between the 116 fall incidents (53%) occurring in the first year of follow-up (57 women) and the 104 fall incidents (47%) in the second year (43 women). Thirty-nine percent (*n *= 31) of the women who sustained a fall during follow-up fell once, 25% (*n *= 20) fell twice, and 35% fell three or more times (*n *= 28).

### Depression and falls

Eighteen percent (*n *= 32) of all women in this study, suffered from depression at baseline (EDS scores > 11), 69% of the depressed women (n = 22) sustained a fall during follow-up. The proportion of women that suffered from depression at baseline and sustained a fall during follow-up (22 out of 79; 28%) was significantly higher than the proportion of women with depression that did not sustain a fall during follow-up ((10 out of 102; 10%); *Χ*^2 ^= 8.76, df = 1, *p *= .003; Table 2). Depression at baseline was not related to the number of falls (once or more during the follow-up period) nor to the time of falling (first- and/or second-year, data not shown).

### Other fall-related risk factors

The prevalence of co-morbidity at baseline was also significantly higher in women who sustained a fall during follow-up compared to those who did not (*Χ*^2 ^= 9.10, df = 1, *p *= .003; Table 2). With respect to the other fall-related risk factors (living on one's one, ≥1 fall during 12 months prior to inclusion, use of a walking aid, use of anti-depressants, use of sedatives, use of antihypertensives, ≥2 units of daily alcohol consumption, and physical inactivity) no significant differences were found between women who sustained a fall during follow-up and women who did not.

### Correlations

As showed in Table 3, significant correlations were found between age and femoral neck BMD, with a decrease in BMD with increasing age (*r *= -.312, *p *=< .001), and femoral neck BMD and lumbar spine BMD with an increase in BMD of the lumbar spine with increasing BMD of the femoral neck (*r *= .370, *p *=< .001).

**Table 3:** Correlations between depression, age, BMD of the femoral neck and BMD of the lumbar spine

Variable	Depressive symptoms	Age	BMD femoral neck	BMD lumbar spine
Depressive symptoms	1	.129	-.039	.071
Age	.129	1	-.312*	-.070
BMD femoral neck	-.039	-.312*	1	.370*
BMD lumbar spine	.071	-.070	.370*	1

* *p *< .001

### Logistic regression analyses

The results of single logistic regression analyses are shown in Table 4. Higher age (OR = 1.06, 95% CI = 1.01-1.11), presence of co-morbidity (OR = 2.68, 95% CI = 1.45-4.97), use of sedatives (OR = 2.53, 95% CI = 1.01-6.38), and depression according to the EDS (OR = 3.55, 95% CI = 1.57-8.04), were found to be significantly related to future falls. Multiple logistic regression showed that the presence of co-morbidity (OR = 2.25, 95% CI = 1.11-4.56) and depression (OR = 4.13, 95% CI = 1.58-10.80) at baseline were independently related to future falls (Table 5). According to logistic regression analyses, no other significant relations were found.

**Table 4:** Single logistic regression, dependent variable: sustaining future falls during the two-year follow-up period in 181 women with a recent low-energy fracture

Variable at baseline	OR	95% CI
Age*	**1.06**	**1.01 - 1.11**
Married/living together	0.71	0.38 - 1.31
BMD femoral neck hip	0.17	0.01 - 2.89
Living on one's own	1.33	0.71 - 2.48
History of falls during 12 months prior to inclusion	1.65	0.78 - 3.48
Use of walking aid	0.96	0.43 - 2.17
Comorbidity^a^*	**2.68**	**1.45 - 4.97**
Use of anti-depressants	2.23	0.52 - 9.63
Use of sedatives*	**2.53**	**1.01 - 6.38**
Use of antihypertensives	0.89	0.47 - 1.66
≥2 units of daily alcohol consumption	1.15	0.57 - 2.32
Physical inactivity	1.60	0.73 - 3.51
Depression according to the EDS^b^*	**3.55**	**1.57 - 8.04**

* *p *< .05

^a ^history of stroke, urinary incontinence, osteoarthritis, rheumatic disease, diabetes and Parkinson's disease

^b ^score > 11

**Table 5:** Multiple logistic regression, dependent variable: sustaining future falls during the two-year follow-up period in 181 women with a recent low-energy fracture

Variable at baseline	Adjusted OR	95% CI
Age	1.02	0.96 - 1.09
Married/living together	0.38	0.04 - 16.54
BMD femoral neck hip	0.19	0.01 - 6.28
Living on one's own	1.05	0.05 - 21.61
History of falls during 12 months prior to inclusion	1.70	0.71 - 4.07
Use of walking aid	0.47	0.17 - 1.29
Comorbidity^a^*	**2.25**	**1.11 - 4.56**
Use of anti-depressants	1.59	0.23 - 11.12
Use of sedatives	2.41	0.79 - 7.37
Use of antihypertensives	1.09	0.53 - 2.22
≥2 units of daily alcohol consumption	1.27	0.57 - 2.83
Physical inactivity	1.47	0.60 - 3.57
Depression according to the EDS^b^*	**4.13**	**1.58 - 10.80**

* *p *< .05

^a ^history of stroke, urinary incontinence, osteoarthritis, rheumatic disease, diabetes and Parkinson's disease

^b ^score > 11

## Discussion

This study shows that the presence of depression after a recent low-energy fracture was an independent risk factor for future falls during a two-year follow-up period in older post-menopausal women (OR = 4.13, 95% CI = 1.58-10.80). Moreover, the existence of co-morbidity increased the risk of falls within two years (OR = 2.25, 95% CI = 1.11-4.56).

Depression at baseline (EDS scores > 11) was present in 18% of the women, which is comparable to other studies carried out in the Netherlands. Beekman *et al*. [17] reported a prevalence of 17% in a general population of Dutch women aged over 60 years, while another Dutch primary care study reported a prevalence of 19% [18].

The one-year incidence of falls among community-dwelling elderly has been estimated at 30%, which is in accordance with our findings (31%) [1]. The number of falls was equally distributed over the two-year follow-up period. However, 72% of the women who sustained a fall during follow-up, fell during the first year of follow-up, while 28% sustained their first fall in the second year. Therefore, the number of falls did not decline over time, but the incidence of women with a new, first fall did. This means that the women who sustained their first fall in the first year of follow-up had a high risk of falling during the second year. It has repeatedly been reported in the literature that a history of a previous fall is a particular risk factor for subsequent falls [2,15,31]. However, in the present study, the history of a fall during the 12 months prior to inclusion did not affect the risk of falling during the two-year follow-up period.

In addition, falls did occur significantly and independently more often in women who were depressed at baseline compared to non-depressed women. In a large sample of older women from the general population (with no recent history of low-energy fracture), Whooley *et al*. [16] showed that depression and falls were independently related during two years of follow-up (OR 1.4). Stalenhoef *et al*. [15] showed that the risk for falling was about twice as high in depressed primary care patients (with no history of a recent low-energy fracture) compared to non-depressed subjects.

The presence of co-morbid conditions at baseline (*e.g*., osteoarthritis, diabetes, Parkinson's disease) was also significantly related to falls during the two-year follow-up. Most of these conditions interfere with normal balance and physical activity and are well known risk factors for falling [1].

Sustaining a fracture can have a major impact on daily life [32], which can result in depression. In turn, depression after a fracture has been shown to be a risk factor for delayed recovery [19,20,33]. Moreover, this study points out that depression is an important risk factor for future falls in women with a recent low-energy fracture, thereby substantially increasing the risk of subsequent fractures. BMD was not related to the incidence of falls. However, it has repeatedly been reported that fractures after falls are particularly common in patients with osteopenia [34,35].

The question why depression is related to falls remains to be answered. It has been shown that the use of anti-depressants and sedatives can lead to falls and hip fracture [2,36,37]. In our study, the use of sedatives was related to falls, only at a univariate level. Haagstra et al. [38] found that health-related quality of life decreased with the number of comorbid diseases in injured patients. It might be argued that the presence of comorbidity affects quality of life in older women with a recent fracture, making them more vulnerable for depression and in the same time more prone to falls through difficulties with walking and locomotion. More research is needed in explaining the relationship between depression and falls and the role of comorbidity.

Several limitations of the present study should be mentioned. Firstly, a bias could have occurred due to the high non-response rate or the use of subjects from only two hospitals from the same area. Depressed women may have been more willing to participate in our study than non-depressed women. Although we have no detailed data on the characteristics of the non-responders, we do know that there were no significant differences between the demographic characteristics (which are important general determinants of depression) of our sample and those of the total population that visited the F&O clinics. Moreover, the number of women with depression according to the EDS and the number of falls occurring in our study were similar to that of the general postmenopausal population, again suggesting no bias. Secondly, we did not assess depression at a syndromic level, which would usually be performed during a structural interview. During such an interview, there is also the opportunity to assess lifetime history of depression and/or chronic episodes of depression. It would be interesting to discover whether women with a previous history of depression are particularly at risk for another depressive episode after a low-energy fracture, and hence for the incidence of future falls. Thirdly, we assessed falls annually by means of detailed questionnaires and interviews, as opposed to using a diary. This may have led to an underestimation of the number of falls. A further limitation is that we did not include other known determinants of falls (*e.g*., vision disabilities, normal daily activities, dizziness).

## Conclusions

In regular outpatient F&O clinics, most clinicians concentrate on the presence of skeletal-related risk factors for fractures (osteoporosis, family history of hip fracture, glucocorticoid use) rather than on risk factors associated with future falls. Depression is an important risk factor for future falls and is associated with delayed recovery. Based on the findings from the present study, we propose that clinicians who are treating patients with recent fractures should anticipate the presence of depression. Further research is needed to evaluate the effect of treatment and the prevention of depression after a recent fracture.

## Competing interests

The authors declare that they have no competing interests.

## Authors' contributions

MvdB is the principle investigator and the main author of the manuscript. VJMP, JPWvdB and GLL are the supervisors of the principle investigator and responsible for the medical and scientific content of the manuscript. NAV, CJMW-vG and ACR have contributed substantially to the intellectual content of the manuscript. All authors have made substantially contributions to conception and design of the study and interpretation of the data or acquisition of data. Furthermore, all authors have been involved in drafting the manuscript or revising it critically for important intellectual content and read and approved the final manuscript and agree with publication of their names.

## Pre-publication history

The pre-publication history for this paper can be accessed here:

http://www.biomedcentral.com/1471-2318/11/73/prepub
